# LET’S PLAY TOGETHER: INTERGENERATIONAL EDUCATION BY CO-DESIGN ESCAPE GAME WITH OLDER ADULTS

**DOI:** 10.1093/geroni/igac059.1714

**Published:** 2022-12-20

**Authors:** Li-Chuan Liu

**Affiliations:** National Taitung University, Taitung, Taitung, Taiwan (Republic of China)

## Abstract

People always think that the older adults are only suitable for participating in activities and games suitable for older adults, and cannot complete games with young people, such as escape game. However, if we can understand the characteristics and needs of the elderly, we find that the older adults still have mobility and vitality to participate in games with young people. Through the intergenerational learning of university’s students and the older adults, we use design thinking and PDCA as class frameworks, and let two groups can jointly together to design escape game for the older adults. During the planning stage, the survey and interview of older adults, game design training; the practical operation in the implementation stage; the team discussion and correction in the reflection stage; and the sharing with older adults in the re-action stage (celebration). As a result, these processes allow students and older adults to communicate and share life experiences and wisdom. In one hand, students have a better understanding and concerns about older adults, and they find that the older adults also have vitality and enthusiasm; on the other hand, older adults also more understand the life of students, and feel the creativity and investment of students. In other words, both groups have changed their cognition to each other, and discovered the possibility of mutual cooperation and communication.

